# Genes Involved in the Transcriptional Regulation of Pluripotency Are Expressed in Malignant Tumors of the Uterine Cervix and Can Induce Tumorigenic Capacity in a Nontumorigenic Cell Line

**DOI:** 10.1155/2019/7683817

**Published:** 2019-12-01

**Authors:** Graciela Ruiz, Heriberto A. Valencia-González, Delia Pérez-Montiel, Felipe Muñoz, Rodolfo Ocadiz-Delgado, Jorge Fernández-Retana, Carlos Pérez-Plasencia, Osbaldo Reséndis-Antonio, Patricio Gariglio, Alejandro García-Carrancá

**Affiliations:** ^1^Departamento de Genética y Biología Molecular, Centro de Investigación y de Estudios Avanzados, Instituto Politécnico Nacional, Mexico City, Mexico; ^2^Programa de Maestría y Doctorado en Ciencias Bioquímicas, Facultad de Química, Universidad Nacional Autónoma de México, Mexico City, Mexico; ^3^Departamento de Patología, Instituto Nacional de Cancerología, Secretaría de Salud, Mexico City, Mexico; ^4^Human Systems Biology Laboratory, Instituto Nacional de Medicina Genómica & Coordinación de la Investigación Científica, Red de Apoyo a la Investigación-Universidad Nacional Autónoma de México, Mexico City, Mexico; ^5^Department of Natural Science, Universidad Autonoma Metropolitana, Cuajimalpa, Mexico City, Mexico; ^6^Dirección de Ingeniería en Nanotecnología y Biotecnología, Universidad Politécnica del Valle de México, Tultitlán, Mexico; ^7^Unidad de Investigación Biomédica en Cáncer, Facultad de Estudios Superiores Iztacala, Universidad Nacional Autónoma de México & Instituto Nacional de Cancerología, Secretaría de Salud, Mexico City, Mexico; ^8^Unidad de Investigación Biomédica en Cáncer, Instituto de Investigaciones Biomédicas, Universidad Nacional Autónoma de México & Instituto Nacional de Cancerología, Secretaría de Salud, Mexico City, Mexico

## Abstract

Transcription factors OCT4, SOX2, KLF4, C-MYC, and NANOG (OSKM-N) regulate pluripotency and stemness, and their ectopic expression reprograms human and murine fibroblasts that constitute the key of regenerative medicine. To determine their contribution to cell transformation, we analyzed the gene expression profiles of these transcription factors in cervical cancer samples and found that they are preferentially expressed in the tumor component. Also, cancer stem cell-enriched cultures grown as sphere cultures showed overexpression of OSKM-N genes. Importantly, we observed that lentiviral-mediated transduction of these factors confers, to a nontumorigenic immortalized human cell line, properties of cancer stem cells as the ability to form tumors in a mouse model. When we performed a meta-analysis using microarray data from cervical cancer biopsies and normal tissues, we found that the expression of OSKM-N and some target genes allowed separating tumor and normal tissues between samples, which enhanced the importance of OSKM-N in the tumorigenesis. Finally, we analyzed and compared both transcript and protein expression profiles of these factors within a cohort of patients with cervical cancer. To our knowledge, this is the first time that the expression of OSKM-N is described to induce one of the main characteristics of the cancer stem cell, the tumorigenicity. And, more importantly, its exogenous expression in a nontumorigenic cell line is sufficient to induce a tumorigenic phenotype; furthermore, the differential expression of this transcription factor distinguishes tumor tissue and normal tissue in cervical samples.

## 1. Introduction

The pluripotency transcription factors OCT4, SOX2, KLF4, C-MYC, and NANOG (OSKM-N) confer properties of stem cells (SC) as self-renewal and pluripotency abilities. It is also known that they form positive feedback regulation, which is involved in the process of cellular self-renewal in SC [1]. Exogenous expression of these genes induces reprogramming of human and mouse adult fibroblasts and confers a stemness and pluripotency phenotype [2–4]. However, it has recently been proposed that these genes also play a role in the pathogenesis of cancer, such as melanoma [5–7], ovarian [8–11], and oral [12–15] tumors. In particular, some of these factors have been found expressed in cervical cancer [16–18]. Cervical cancer (CC) remains a major public health problem in the world, with more than 265,000 women dying each year due to this disease [19]. A variable percentage of patients diagnosed with this cancer presents recurrence, up to 90% of cases in less than 3 years after initial treatment [20]. Therefore, it is essential to improve the therapy for this condition. Currently, cancer stem cells are considered responsible for resistance to chemotherapy in CC.

Cancer stem cells (CSC) are distinguished from the bulk of the tumor cells by their unique capacity for self-renewal, maintenance of the malignant phenotype, and the generation of tumors; in addition, they have been proposed as responsible for the metastasis and tumor recurrence [21, 22]. The existence of CSC suggests that normal stem cells undergo mutations that could lead to their transformation. CSC have deregulated homeostatic control over the generation of more CSC; in addition, cancer cells with different phenotypes possess heterogeneous tumorigenic potential [21]. This new concept is changing our understanding of the development and progression of tumors.

The capacity for self-renewal is necessary for the maintenance of CSC as well as generation of the cell progeny in proliferation. Thus, it is vital to study the genes that could be regulating this process and their contribution in carcinogenesis. It has also been proposed that CSC play a central role in resistance to chemotherapy and radiotherapy and that they are responsible for cancer recurrence after treatment, although the majority of cancer cells are eliminated [23]. Recently, it was possible to obtain CSC-enriched cultures from established cell lines derived from cervical cancer [24]. These cell cultures were grown as spheres under nonadherent conditions and were positive for the cell surface protein CD49f, in addition to the expression of CSC-specific markers such as OCT4, SOX2, NANOG, and Annexin II [25].

To determine the contribution of OSKM-N in the tumorigenic process and the induction of CSC, we analyzed their expression in tumoral samples from CC and in CSC-enriched cultures grown as spheres. We transduced these factors in a nontumorigenic human cell line to observe whether they can confer a tumorigenic phenotype. We also utilize an algorithm to perform bioinformatics analysis of all the previously published data obtained from microarray assays that compared tumoral samples with normal tissues. These data were used to identify pioneer transcription factors and to differentiate, *in silico*, normal tissue from tumoral tissue. Importantly, using immunohistochemistry, we observed that variations in the expression levels of OSKM-N genes were specifically associated with the tumor cells in CC patients and are part of a genetic signature characteristic of CSC in this type of tumor.

## 2. Materials and Methods

### 2.1. Patient Samples

Twenty-two cervical cancer biopsies and one hysterectomy sample with nonmalignant or nontumor tissue diagnosis, available at the laboratory's tumor bank, were kept frozen at -80°C and used for western blot (WB) analysis. Twenty-two paraffin blocks of cervical tumor samples were included in this study; cuttings of 5 *μ*m thickness were made, collected, and fixed on electrocharged slides for further analysis by immunohistochemistry and histopathological studies.

### 2.2. Protein Extraction from Cervical Cancer Biopsies

Frozen tumor samples were ground on liquid nitrogen. Total protein was extracted using lysis buffer (50 mM Tris-base, 5 mM EDTA, 133 mM NaCl, 1 mM PMSF, and 1% Triton X-100) that contains protease inhibitors (complete inhibitor tablet, Roche) and a 22G needle for cell lysing. Quantification was performed using the Pierce™ BCA Protein Assay kit (Thermo Scientific). The already extracted proteins were preserved at -80°C.

### 2.3. Cell Lines

We employed HeLa and SiHa cell lines, which are derived from cervical cancer and contain HPV types 18 and 16, respectively. We used the HaCaT cell line as nontumorigenic cells, which is an immortalized human keratinocyte cell line. HeLa, SiHa, and HaCaT cell lines were grown in Dulbecco's modified Eagle's medium (DMEM) (Gibco) supplemented with 10% fetal bovine serum (FBS) (Gibco), 100 *μ*g/mL streptomycin, and 100 U/mL benzylpenicillin (Gibco). These cell lines were grown at 37°C in a humidified atmosphere containing 5% CO_2_. The experiments were performed when the cultures were between 70% and 80% of confluence. Cell lines were authenticated by STR DNA profiling at the University of Colorado DNA Sequencing and Analysis Core according to the report number DP0297 issued by them. We used the HEK 293T cell line derived from the human embryonic kidney to obtain lentiviral particles; this cell line was grown in DMEM supplemented with 10% FBS at 37°C in a humidified atmosphere containing 5% CO_2_.

### 2.4. Sphere-Forming Assay

HeLa, SiHa, and HaCaT cells were seeded under nonadherent conditions to obtain cancer stem cell-enriched cultures. We used MammoCult™ medium (Stem Cell Technologies) with serum replacement, hydrocortisone, heparin, and antibiotics according to the supplier's instructions (Stem Cell Technologies) in 100 mm ultralow attachment plates at a density of 3,000 cells/mL growing for 7 days at 37°C in a humidified atmosphere containing 5% CO_2_. For analysis of sphere formation, 100 cells per well were plated in 96-well culture dishes with 200 *μ*L of growth medium. The number of spheres for each well was evaluated 7 days after seeding, and sphere formation rate was counted. Sphere-forming efficiency (SFE) was calculated as the number of spheres formed divided by the original number of single cells seeded and expressed as a percentage.

### 2.5. Generation of Lentiviral Particles

A cotransfection was performed with the plasmids Pax2 and PDM2 together with the gene of interest: OCT4, SOX2, KLF4, C-MYC, or NANOG in HEK-293T cells. Lentiviral particles were harvested 72 hours posttransfection. They were aliquoted and frozen at -80°C until use.

### 2.6. Addgene Plasmids

The plasmids employed to generate lentiviral particles were OCT4 (pSin-EF2-OCT4-Pur was a gift from James Thomson; Addgene plasmid # 16579), SOX2 (pSin-EF2-SOX2-Pur was a gift from James Thomson; Addgene plasmid # 16577), KLF4 (FUW-tetO-hKLF4 was a gift from Rudolf Jaenisch; Addgene plasmid # 20725), C-MYC (FUW-tetO-hMYC was a gift from Rudolf Jaenisch; Addgene plasmid # 20723), NANOG (pSin-EF2-NANOG-Pur was a gift from James Thomson; Addgene plasmid # 16578), OSKM (TetO-FUW-OSKM was a gift from Rudolf Jaenisch; Addgene plasmid # 20321), PM2G (pMD2.G was a gift from Didier Trono; Addgene plasmid # 12259), EGFP (PL-SIN-EF1*α*-EGFP was a gift from James Ellis; Addgene plasmid # 21320), and Pax2 (psPAX2 was a gift from Didier Trono; Addgene plasmid # 12260) [4, 26, 27].

### 2.7. Gene Transduction

Stable transfections were performed on HaCaT cells utilizing the lentiviral particles to transduce OCT4, SOX2, KLF4, C-MYC, or NANOG proteins and selected with puromycin. Lentiviruses were used with the EGFP protein to be able to evaluate the infectivity of viral particles by microscopy and flow cytometry. To obtain stable clones, 285 ng/mL puromycin (Gibco) was used during 30 days for selection.

### 2.8. Cell Transfection

OSKM-N overexpression sequence was inserted into the pcDNA 3.1 vector. Cells were transfected with 2 *μ*g pcDNA3.1-OSKM-N using a Lipofectamine 3000 Transfection Kit (Invitrogen, USA) according to the manufacturer's instructions. Cells were collected at 72 h posttransfection for further experiments. Cells transfected with the pcDNA3.1 empty vector were considered the empty vector group.

### 2.9. Quantification of mRNA Transcription Levels

Reverse transcriptase-quantitative polymerase chain reaction (RT-qPCR) assays were performed to evaluate the messenger RNA (mRNA) levels of OCT4, SOX2, and NANOG in HeLa and SiHa spheres and their monolayer counterparts. Total RNA extraction was performed using the TRIzol reagent (Invitrogen). RNA quality and quantification were determined employing the Epoch™ Take3 microplate spectrophotometer (BioTek, UK). RNA was digested with DNAse I (Biolabs, UK) and utilized for complementary DNA (cDNA) synthesis with the first-strand synthesis system SuperScript III system (Invitrogen, USA). RT-qPCR was performed by adding 500 ng of cDNA, 10 *μ*L of SYBR Green mix, 100 ng of specific primers, and water to obtain a final volume of 20 *μ*L. Quantitative real-time PCR was performed with an Applied Biosystems 7300 system (Foster City, CA, USA), and we used StatMiner™ Real-Time software to obtain the results and the delta-delta C_T_ method to perform the statistical analysis. The beta2-microglobulin (*β*2M) gene was used as an internal control. Oligos designed to amplify OCT4, SOX2, and NANOG were as follows: OCT4 (F) 5′TGCGTCACACCATTGCTATTCTTC3 ′, (R) 5'CTTAGCCAGGTCCGAGGAT3′; SOX2 (F) 5′TGCGTCACACCATTGCTATTCTTC3 ′, (R) 5′CTTCGGATTTCGCCTTCTC3 ′; NANOG (F) 5′CTTCGGATTTCGCCTTCTC3 ′, (R) 5′TGCGTCACACCATTGCTATTCTTC3 ′; and *β*2M (F) 5′ACCCCCACTGAAAAAGATGAG3 ′, (R) 5′ATGATGCTGCTTACATGTCTCG3 ′.

### 2.10. Immunodetection of Proteins by Western Blot

60 *μ*g of total protein was separated on a 10% SDS-PAGE gel according to standard procedure, and the transfer was performed on a 0.45 *μ*m nitrocellulose membrane (Thermo Scientific); the membrane was blocked with 5% low-fat milk for 1 h. Subsequently, the membranes were incubated with the different antibodies: anti-OCT4 (Santa Cruz Biotechnology, sc-5279), anti-SOX2 (Santa Cruz Biotechnology, sc-365823), anti-KLF4 (Thermo Fisher Scientific, MA5-15672), anti-C-MYC (Santa Cruz Biotechnology, sc-40), and anti-NANOG (Santa Cruz Biotechnology, sc-376915). Then, the membranes were incubated with an anti-mouse secondary antibody (Santa Cruz Biotechnology, sc-2005). As a loading control, *β*-ACTIN (Santa Cruz Biotechnology, sc-47778) was used. For detection of the protein-antibody complex, we used the Immobilon Western Chemiluminescent HRP Substrate (Millipore, WBKLS0500) according to the manufacturer's specifications and visualized it with C-DiGit equipment (Li-Cor). Data were presented as relative expression protein levels normalized for *β*-ACTIN protein.

### 2.11. Tumor Formation in NOD-SCID Mice

Nonobese diabetic-severe combined immunodeficiency (NOD-SCID) female mice aged 4-6 weeks were used. HaCaT cells were transduced with OCT4, SOX2, KLF4, C-MYC, or NANOG to induce tumorigenic capacity. In brief, 6 × 10^6^ cells were inoculated subcutaneously into one of the lower flanks of each mouse in a group of six mice to assess whether HaCaT cells acquired the ability to form a tumor. In a second set of six mice, 4 were inoculated with 6 × 10^6^ and 2 mice with 8 × 10^6^ nontransduced HaCaT cells. A third group of six mice was inoculated with 2 × 10^6^ HeLa cells. As a carrier, during the inoculation, DMEM medium was used in a volume of up to 200 *μ*L. Tumor growth was followed for 4 weeks for HeLa and 8 weeks for HaCaT. Mice euthanasia was performed in a CO_2_ chamber.

### 2.12. Immunohistochemistry and Immunocytochemistry

Histological sections of 5 *μ*m were obtained from paraffin blocks of patients with CC. Hematoxylin-eosin staining was performed to confirm the histopathological diagnosis. Transduced HaCaT cells and selected by puromycin were seeded in slides, and together with histological sections of CC were stained with OCT4, SOX2, KLF4, C-MYC, NANOG, and P16^INK4a^ antibodies. Immunohistochemistry and immunocytochemistry were performed by BenchMark GX IHC/ISH system Ventana 750-800 (Roche, USA).

### 2.13. Bioinformatic Analysis of Microarrays

It used the Gene Expression Omnibus database, which considers all of the cDNA microarrays reported by the Affymetrix platform. Reported cDNA microarrays employed samples from the HeLa cell line, the HaCaT cell line, cervical cancer, and cervical normal tissue biopsies. Also, the microarray data of Gene Expression for 85 biopsies is available at the GEO (Gene Expression Omnibus database) (https://www.ncbi.nlm.nih.gov/geo/), with access number GSE56303.

### 2.14. Ethical Considerations

All methods were carried out in accordance with relevant guidelines and regulations and approved by the Committee of Research Ethics of Instituto Nacional de Cancerología (INCan, Mexico), which is registered in the Office for Human Research Protections. The approval number is (017/049/IBI) (CEI/1164/17).

We included samples of frozen tumors from patients available in our laboratory and paraffin blocks from tumor samples from patients of the pathology service of the institute. We analyzed 85 samples from patients whose clinical outcome was known after treatment [28]. Samples that suffered from thawing or paraffin blocks belonging to patients who have abandoned treatment were excluded. No new fresh samples were collected from patients. The laboratory mice were acquired from the “Unidad de Modelos Biológicos of Instituto de Investigaciones Biomédicas (IIB) de la Universidad Nacional Autónoma de México (UNAM, Mexico)”, and the handling and experimentation of the mice were carried out in the facilities for animal of INCan in accordance with local guidelines such as the official Mexican standard NOM-062-ZOO-1999, the code of ethics of IIB-UNAM, and other international guidelines [29].

### 2.15. Statistical Analysis

The quantitative data were expressed as the mean ± standard deviation (SD); in at least 3 independent experimental replicates, the qualitative data were expressed as a percentage or in proportion. The Student *t*-test was performed to evaluate the significance of the expression of each of the OCT4, SOX2, NANOG, KLF4, and C-MYC genes between normal tissues and tumors. It was considered that there is a statistically significant difference when the *p* value obtained in the analyses was smaller than 0.05. The GraphPad Prism ver.6 for Windows statistical software package was employed in all analyses.

## 3. Results

### 3.1. Cervical Cancer Biopsies Express High Levels of OSKM-N Pluripotency Transcription Factors

**Figure 1:**
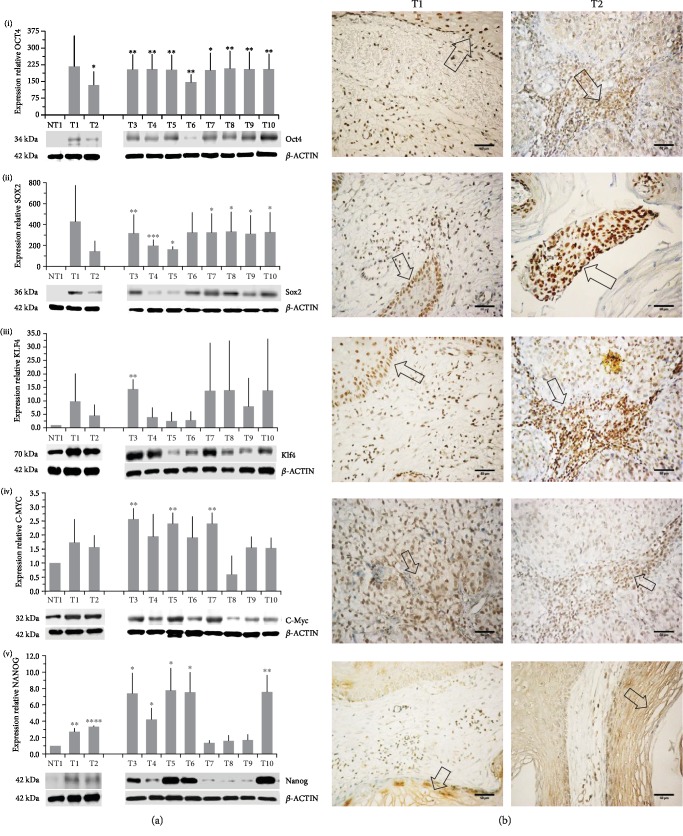
OSKM-N proteins are expressed in cervical cancer. (a) Expression levels of OCT4 (i), SOX2 (ii), KLF4 (iii), C-MYC (iv), and NANOG (v) proteins in cervical cancer tumors were measured by western blot (WB). Tumor samples (T1-T10) were compared with a nontumor sample (NT1). WB experiments were performed in triplicate; the values are expressed as mean ± standard deviation (normalized to *β*-actin) using densitometric analysis. (b) The expression of OSKM-N in tumor cells from T1 and T2 samples was observed by immunohistochemistry (arrows). ×20 magnification. ^∗^*p* < 0.05, ^∗∗^*p* < 0.01, ^∗∗∗^*p* < 0.001, ^∗∗∗∗^*p* < 0.0001.

**Figure 2:**
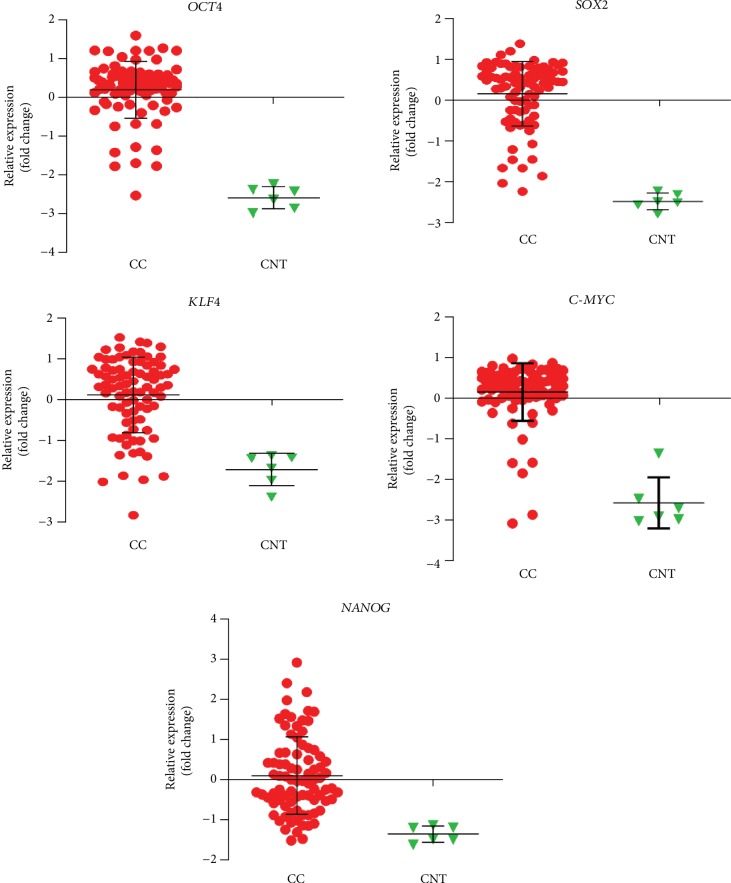
Differential expression of stemness- and pluripotency-related genes in tumor biopsies and normal tissues. The scatter plots illustrate data from 85 cervical cancer and 6 normal samples grouped by tumor and normal tissue. The gene expression intensity values were obtained by microarray analyses for *OCT4*, *SOX2*, *KLF4*, *C-MYC*, and *NANOG*. Patients were grouped by cervical cancer (CC) biopsies and cervical normal tissue (CNT).

The level of expression of OCT4, SOX2, KLF4, C-MYC, and NANOG (OSKM-N) proteins was higher in cervical cancer (CC) samples than in a nontumor sample. OCT4 and SOX2 were found expressed significantly higher in 9/10 and 7/10 tumor samples, respectively (Figure 1(a)). Only some samples expressed significantly higher level of NANOG (7/10), KLF4 (1/10), and C-MYC (3/10) than nonmalignant tissue (Figure 1(a)). We analyzed the significance among normal and tumor tissues, and we found that only OCT4 (*p* value < 0.0001) and SOX2 (*p* value = 0.0014) had higher expression in tumor tissues, not so for KLF4 (*p* value = 0.0610), C-MYC (*p* value = 0.0900), or NANOG (*p* value = 0.0969). Also, the OSKM-N proteins were expressed at high levels in the tumor component of the CC samples (Figure 1(b)). Then, we found that the expression of the OSKM-N genes is higher in 85 CC samples than in 6 normal cervical tissues (Figure 2). In addition, when these samples were grouped by clinical outcome, we found similar results ([Supplementary-material supplementary-material-1]).

### 3.2. Cancer Stem Cell-Enriched Cultures Overexpress OSKM-N Compared to Monolayers of Cervical Cancer Cell Lines

**Figure 3:**
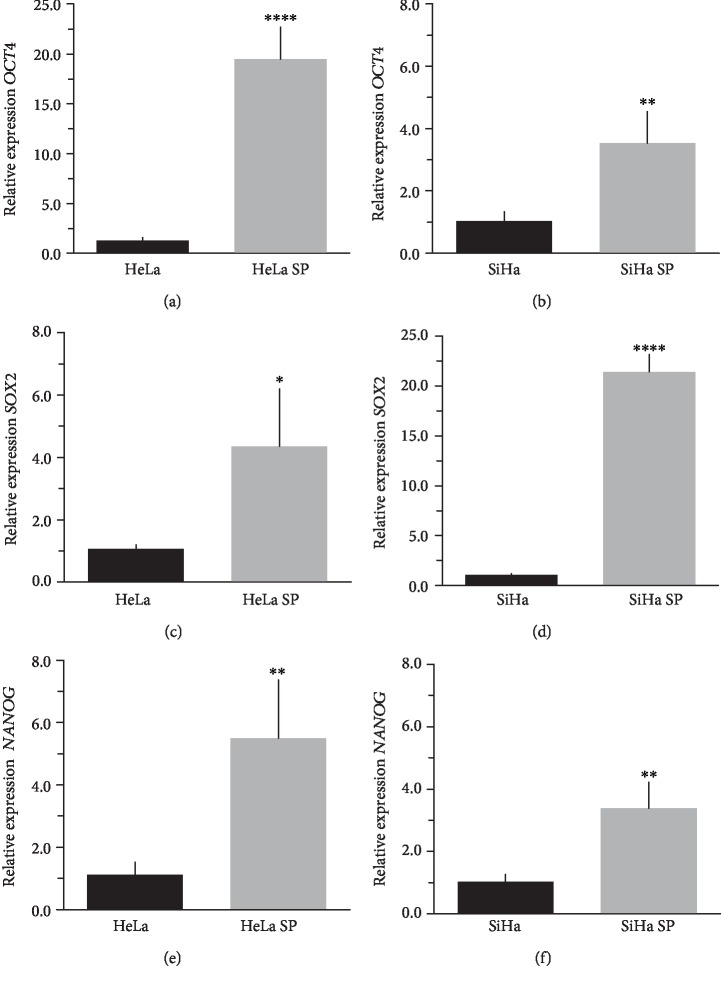
The mRNA levels of *OCT4*, *SOX2*, and *NANOG* are high in cancer stem cell-enriched cultures. The messenger RNA levels of *OCT4*, *SOX2*, and *NANOG* are higher in the cancer stem cell-enriched cultures grown as spheres from HeLa (HeLa SP) and SiHa (SiHa SP) cells than in their monolayer cultures (HeLa and SiHa, respectively) grown conventionally. Experiments were performed in triplicate, and the values are expressed as mean ± standard deviation. Beta2-microglobulin (*β*2M) was used as the housekeeping gene. ^∗^*p* value < 0.05, ^∗∗^*p* value < 0.01, and ^∗∗∗∗^*p* value < 0.0001.

The literature describes the OSKM-N factors as markers of normal pluripotent stem cells. However, in CC, it has not yet been elucidated whether CSC express these factors. In our group, previously, we have characterized and isolated a subpopulation of cancer stem cells from cell lines established from CC (HeLa, SiHa, Ca Ski, and C-4 I) using the sphere-forming assay and found they express characteristic markers of stem cell. In addition, these subpopulations were able to generate tumors with 10-100 times less cells than monolayer cultures [24]. Also, we have reported an extended phenotype for cervical cancer stem cells that showed an increased activity of enzyme aldehyde dehydrogenase (ALDH) as well as a higher expression of CK-17, p63, Annexin II, and CD49f in cancer stem cell-enriched cultures from cervical cancer. Interestingly, the high ALDH activity correlated with higher tumorigenic activity [25] and exhibited increased radioresistance [30]. We analyzed their expression using RT-qPCR and found that OCT4, SOX2, and NANOG are overexpressed in CSC-enriched sphere cultures as compared with same cell lines grown under adherent conditions from HeLa (Figures 3(a), 3(c), and 3(e)) and SiHa (Figures 3(b), 3(d), and 3(f)) cell lines derived from cervical cancer. In a previous work in our laboratory, Ortiz-Sánchez et al. evaluated the expression of OCT4 and NANOG using flow cytometry [25].

### 3.3. OSKM-N Pioneer Transcription Factors Induce the Formation of CSC-Enriched Cultures in the HaCaT Cell Line

**Figure 4:**
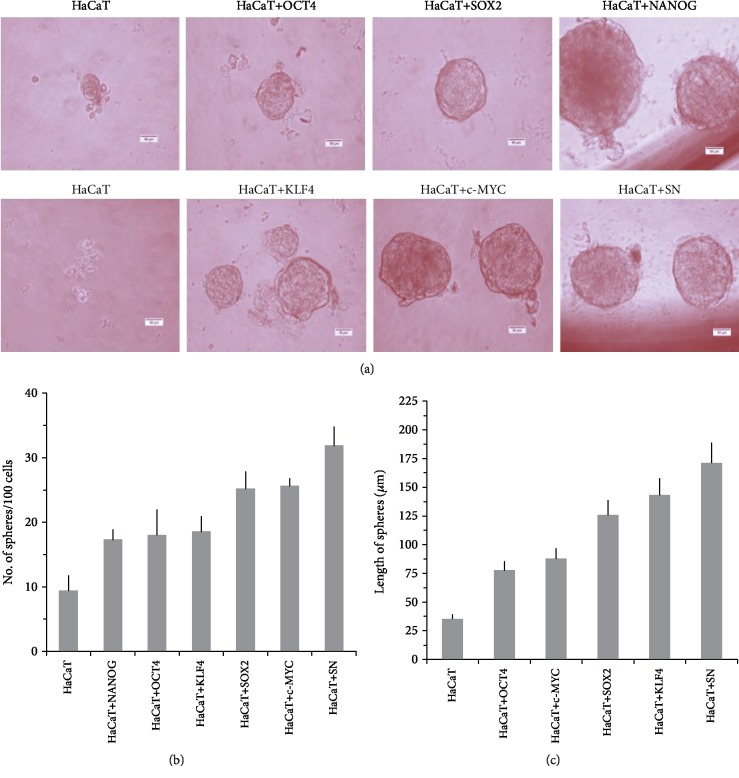
HaCaT cell line increases the efficiency of sphere formation in the presence of the OSKM-N factors. OSKM-N gene overexpression enhances cancer stem cell properties in HaCaT cells. HaCaT cells were cotransfected with OSKM-N gene plasmids and cultured in sphere-forming media. (a) Spheres formed by HaCaT cells compared with HaCaT transfected with OCT4, SOX2, KLF4, C-MYC, NANOG, and a combination of SOX2 plus NANOG (SN). (b) Efficiency of sphere formation of OSKM-N gene-transfected HaCaT cells and (c) length of sphere. The scale bar is 50 *μ*m. Experiments were performed in triplicate, and the values are shown as the mean ± standard deviation. ×40 magnification.

2Overexpression of OSKM-N factors in CSC-enriched populations permitted us to think that, if these genes are overexpressed in the nontumorigenic cell line HaCaT, it would be possible to induce tumorigenicity by forming *in vitro* spheres and the ability to form tumors *in vivo*. We overexpressed OSKM-N factors in HaCaT cell line by transient transfection. After 72 h of transfection, we performed the sphere-forming assay, and after 10 days of culture, we evaluated the formation and size of these spheres (Figure 4(a)). Interestingly, HaCaT cells overexpressing OSKM-N factors individually increased their efficiency of sphere-forming (Figure 4(b)), as well as the size of these spheres (Figure 4(c)) compared with not transfected cells. We obtained the highest efficiency of sphere formation when SOX2 and NANOG (SN) factors were cotransfected.

### 3.4. OSKM-N Factors Induce a Tumor Phenotype in the HaCaT Nontumorigenic Cell Line

To evaluate the induction of tumorigenicity in HaCaT cells by OSKM-N factors, we obtained cells that stably expressed OCT4, SOX2, KLF4, C-MYC, or NANOG by infection with lentiviral particles after the selection period. We corroborated the expression of OCT4, SOX2, and NANOG proteins in the nucleus of HaCaT cells ([Supplementary-material supplementary-material-1]) by immunohistochemistry. In NOD-SCID mice, 6 × 10^6^ of HaCaT cells transduced with OSKM-N were inoculated. HaCaT+OCT4, HaCaT+SOX2, HaCaT+KLF4, HaCaT+C-MYC, HaCaT+NANOG, and HaCaT+OSKM-N (the combination of all the factors) variably formed palpable tumors after 7 days ([Supplementary-material supplementary-material-1]). Up to 8 × 10^6^ HaCaT cells did not develop any tumor while 2 × 10^6^ HeLa cells generated a large tumor as shown in Table 1. The evaluation of infectivity of the lentiviral particles showed that 50% of HeLa cells and HaCaT cells were positive to enhanced green fluorescent protein (EGFP) by microscopy and flow cytometry (Figures [Supplementary-material supplementary-material-1] and [Supplementary-material supplementary-material-1]).

### 3.5. OSKM-N Factors and Other Proteins Are a Signature in Tumor Samples and Segregate Them from Normal Tissue

**Figure 5:**
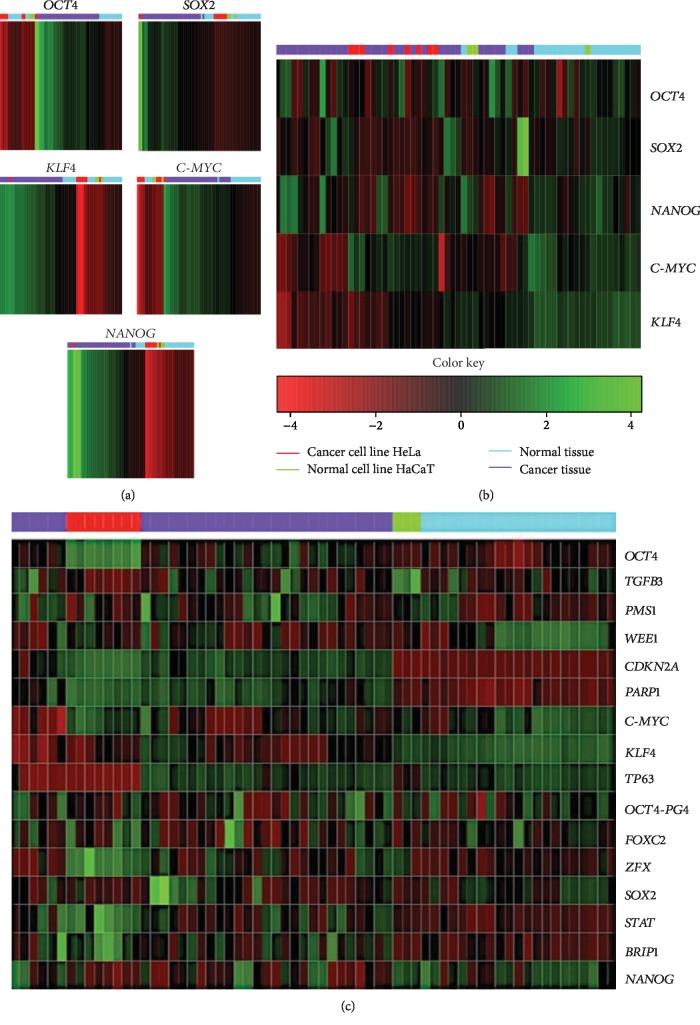
OSKM-N factors segregate tumor cells and nontumor cells from different tissues. The meta-analysis of cDNA microarrays reported for cervical cancer showed a difference in expression profiles between cervical cancer biopsies and normal cervical tissue. (a) The analysis evaluates, through a heat map, each factor separately, (b) using the OSKM-N factors together and (c) OSKM-N factors together with other associated genes regulated by these factors. The color red or green in cells reflects low or high relative expression levels, respectively.

Through a bioinformatic analysis of microarrays using the Gene Expression Omnibus database, which considers all the cDNA microarrays reported by the Affymetrix platform, employing samples from the HeLa cell line, the HaCaT cell line, cervical tumors, and normal cervical tissue biopsies, we found that each transcription factor groups properly in the expression profiles of biopsies obtained from cervical tumors and segregates the normal tissue in another group, both individually (Figure 5(a)) and when they were analyzed together (Figure 5(b)). Even more interesting is that there were other proteins (some of them are targets of OSKM-N factors) which together separate better the tumor population from normal tissue (Figure 5(c)). Proteins such as STAT3, TGF*β*3, LEFTY A, PARP1, and ZFX are also expressed in cervical tumor samples ([Supplementary-material supplementary-material-1]).

### 3.6. OCT4, SOX2, and KLF4 Are Overexpressed in Tumor Cells in Biopsies of Cervical Cancer with Different Clinical Outcomes

**Figure 6:**
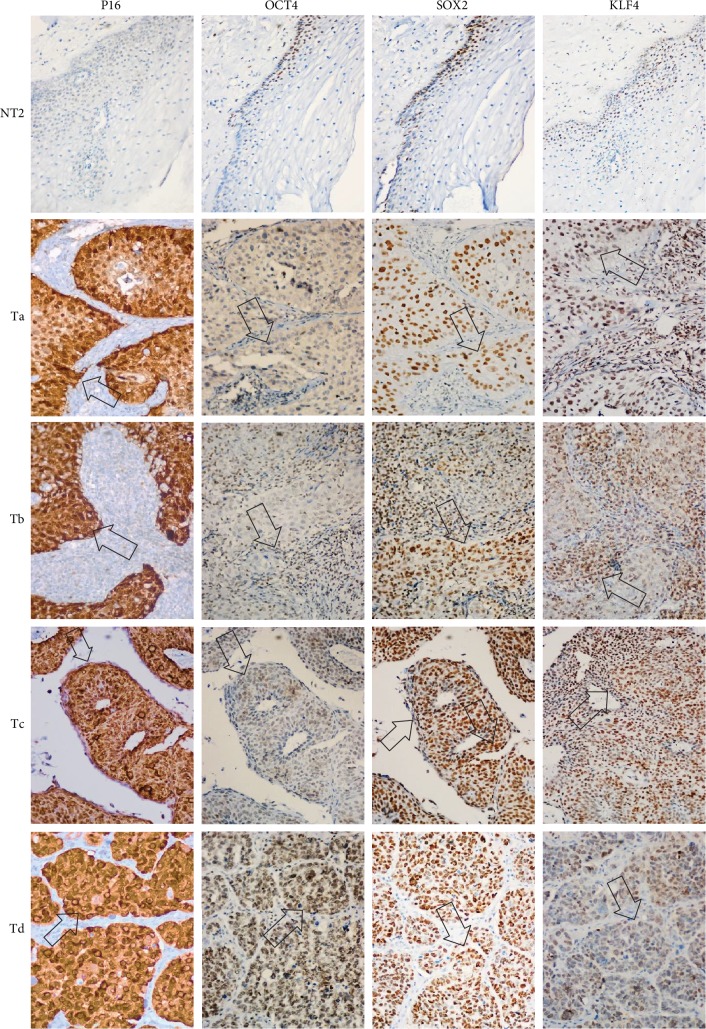
Pluripotency factors are expressed in CC with different clinical outcomes. OSKM-N factors are overexpressed in cervical cancer tumor cells (arrows). P16^INK4A^ was used as an indirect indicator of the presence of HPV and the degree of epithelial injury. The expression of OCT4, SOX2, and KLF4 was examined in 44 CC tissues from which the clinical response to treatment was known. Representative images of four samples are shown. ×40 magnification.

Based on the role of OCT4, SOX2, and KLF4 in tumorigenesis, we decided to evaluate their expression in samples of patients diagnosed with CC in which clinical outcome to the treatment administered to each of them was known. Clinical outcomes were classified as complete or progression/recurrence. We evaluated the expression of OCT4, SOX2, and KLF4, as well as P16^INK4a^, a marker for the presence of HPV [31] by immunohistochemistry (Figure 6). The histopathology of the samples was determined by an expert pathologist. In each sample, nontumoral and tumoral tissue was found, encompassing within the latter the cellular components in *in situ* cancer, invasive cancer, and inflammatory component, elements that were identified in many cases. It is interesting that these factors are overexpressed specifically in the tumoral component. Also, clinical data of 85 patients, diagnosed with locally advanced cervical cancer submitted to standard treatment and followed for 5 years, were obtained as described in Fernandez-Retana et al. [32]. We analyzed for the first time the relative mRNA expression for *OCT4*, *SOX2*, *KLF4*, *C-MYC*, and *NANOG* in tumor and normal cells. Interestingly, we found that the mRNA level of stemness and pluripotency-related genes can distinguish between tumor cells and normal cells as we expected ([Supplementary-material supplementary-material-1]).

## 4. Discussion

The core transcription factors OCT4, SOX2, KLF4, C-MYC, and NANOG (OSKM-N) regulate pluripotency and stemness, and their ectopic expression in human and murine fibroblasts induces pluripotent stem cell formation. It is noteworthy that these genes constitute the mainstay of regenerative medicine due to their ability to give rise to a stemness state and a pluripotent phenotype in adult fibroblasts of mice or human when they are exogenously expressed [2–4]. In this study, we revealed evidence that these factors are differentially expressed in tumor cells derived from cervical cancer samples but not in nontumoral samples. And most importantly, as it has been reported by Zaret and Carroll in the work about pioneer transcription factors [33], our work reveals for the first time new details about the functions and activity of OSKM-N that induce tumorigenicity in a nontumorigenic cell line. The understanding of how the pioneering factors act in the regulation of the tumorigenic process will allow obtaining information about the factors that regulate cell reprogramming and the mechanism that controls carcinogenesis.

Our results demonstrated that OSKM-N factors were preferentially expressed in the tumorigenic cells, which is consistent with previous studies. NANOG has been reported in cervical epithelial lesions [34] and rectal cancer [35] as well as in a group of patients with radioresistant cervical cancer where NANOG was in nucleus [34]; in another study, NANOG induced tumorigenesis *in vivo* [36].

The expression of OCT4, SOX2, and NANOG in cancer stem cell-enriched cultures of HeLa, SiHa, and CaSki cell lines from cervical cancer was higher than their conventional cultures in monolayer. Thereby, to our knowledge, we provide the first proof of concept that there is a potential link between these pioneer transcription factors and cancer stem cell self-renewal.

Exploring the tumorigenic properties of OCT4, SOX2, and NANOG in HaCaT cell line, which did not form tumor *in vivo*, we found that it is possible to obtain tumorigenic cells from nontumorigenic cells, suggesting that they have CSC properties mediated by the presence of these transcriptional factors. Similarly, OCT4, SOX2, and NANOG increased the ability to form spheres indicating a role in obtaining cancer stem cell properties. It is important to note that it has been previously reported that one of the characteristics of stem-like cells is the ability of self-renewal and extensive proliferation as clonal nonadherent spherical clusters [37], expression transcription factors [25], tumorigenic capacity, and radioresistance [25, 30]. This evidence is supported by a report that showed that SOX2 could increase CSC formation in breast cancer cells [38].

In our study, we set out to test the hypothesis that the expression of pioneer transcription factors contributes to cancer by promoting in nontumorigenic cells the acquisition of tumorigenic properties and the acquisition of cancer stem cell properties as the sphere formation. We provide compelling evidence indicating that tumor cells in patient samples express OCT4, SOX2, and NANOG. These factors have already been associated with poor prognosis [39] and poor clinical survival in patients with cervical squamous cell carcinoma [40], breast cancer [41–44], esophageal [45, 46] lung [47] carcinoma and are involved in the cellular transformation towards a malignant phenotype [48–51]. The observation that these pioneer transcription factors were expressed only in the tumor component supports this conclusion.

Also, we show robust results of the expression of OSKM-N proteins in cervical cancer samples with different clinical outcomes, which lead us to suppose that these factors could be therapeutic targets for specifically eliminating cancer stem cells. In this study, we provide new insight into the tumorigenic process in cervical cancer, evidencing that the expression of pioneer factors is sufficient for generating tumorigenic capacity and enhancing properties of cancer stem cells as the sphere and tumor formation. Our data suggest that these factors may hold significant promise as a novel molecular therapy for human cervical cancer, specifically eliminating CSC. Inhibition of important molecules in the tumor development could play a clinical role in cancer treatment, but further studies are needed for medical applications.

## Figures and Tables

**Table 1:** *In vivo* tumorigenic properties of OCT4, SOX2, KLF4, C-MYC, and NANOG.

Condition	Tumor size	Mice with tumor
HeLa	++++	6/6
HaCaT	-	0/6
HaCaT+EGFP	-	0/6
HaCaT+OCT4	+	6/6
HaCaT+SOX2	++	6/6
HaCaT+NANOG	+++	6/6
HaCaT+KLF4	++	6/6
HaCaT+C-MYC	++	6/6
HaCaT+OSKM-N	+++	6/6
HaCaT+SOX2+NANOG	+++	6/6

HaCaT cells were infected with EGFP-, OCT4-, SOX2-, KLF4-, C-MYC-, and NANOG lentiviruses, cultured and puromycin-selected for 1 month, and then injected into NOD-SCID female mice (*n* = 6 for each experimental condition). The + symbol represents positive tumor generation within a period of 7 weeks. HaCaT and HeLa cells were utilized as negative and positive controls, respectively. The results of these experiments indicated that the overexpression of OCT4, SOX2, KLF4, C-MYC, and NANOG taken together was associated with significant tumor growth in HaCaT cells. The number of tumors formed and the number of inoculations performed are indicated for each condition as a fractional number. + represents the size of the tumors: the greater the number of symbols, the larger the tumor size. - represents the absence or nonformation of a tumor.

## Data Availability

The data used to support the findings of this study are included within the article and the supplementary material.

## Supplementary Materials

Supplementary Materials Figure S1 provides information on the expression of OCT4, SOX2, KLF4, C-MYC, and NANOG (OSKM-N) genes in samples from patients with different clinical outcomes and normal tissues. The scatter plots illustrate the data of 85 patients with cervical cancer grouped into progressive disease (PD), recurrent disease (RD), complete response (CR), and normal cervical tissue (CNT). The gene intensity values were obtained by microarray analysis for each gene. Figure S2 shows the infectivity of the lentiviruses, which expressed the green fluorescent protein (EGFP), identified by flow cytometry, in HeLa and HaCaT cells. In addition, after transduction, OCT4, SOX2, and NANOG proteins were detected in HaCaT cells by immunocytochemistry. Figure S3 shows representative images of the tumors formed by injecting 6 × 10^6^ HaCaT cells transduced with OCT4, SOX2, NANOG, C-MYC, or KLF4 individually or all together as OSKM (OCT4, SOX2, C-MYC, and KLF4). HaCaT cells did not form tumors with even 8 × 10^6^ cells. Figure S4 provides data on the expression of proteins associated with OSKM-N factors in cervical cancer, such as STAT3, TGF*β*3, and LEFTY A, which were detected by western blot in 12 cervical tumor samples, as well as PARP1 and ZFX, detected in 8 samples of cervical tumors.
