# Supplementary material for: Genes Involved in the Transcriptional Regulation of Pluripotency Are Expressed in Malignant Tumors of the Uterine Cervix and Can Induce Tumorigenic Capacity in a Nontumorigenic Cell Line
Source: Stem Cells Int. 2019 Dec 1;2019:7683817. doi: 10.1155/2019/7683817 (PMC6914900; doi:10.1155/2019/7683817)
Supplement: Supplementary Materials — Figure S1 provides information on the expression of OCT4, SOX2, KLF4, C-MYC, and NANOG (OSKM-N) genes in samples from patients with different clinical outcomes and normal tissues. The scatter plots illustrate the data of 85 patients with cervical cancer grouped into progressive disease (PD), recurrent disease (RD), complete response (CR), and normal cervical tissue (CNT). The gene intensity values were obtained by microarray analysis for each gene. Figure S2 shows the infectivity of the lentiviruses, which expressed the green fluorescent protein (EGFP), identified by flow cytometry, in HeLa and HaCaT cells. In addition, after transduction, OCT4, SOX2, and NANOG proteins were detected in HaCaT cells by immunocytochemistry. Figure S3 shows representative images of the tumors formed by injecting 6 × 10^6^ HaCaT cells transduced with OCT4, SOX2, NANOG, C-MYC, or KLF4 individually or all together as OSKM (OCT4, SOX2, C-MYC, and KLF4). HaCaT cells did not form tumors with even 8 × 10^6^ cells. Figure S4 provides data on the expression of proteins associated with OSKM-N factors in cervical cancer, such as STAT3, TGFβ3, and LEFTY A, which were detected by western blot in 12 cervical tumor samples, as well as PARP1 and ZFX, detected in 8 samples of cervical tumors. [file 7683817.f1.pdf]

SUPPLEMENTARY FIGURES

Figure S1.

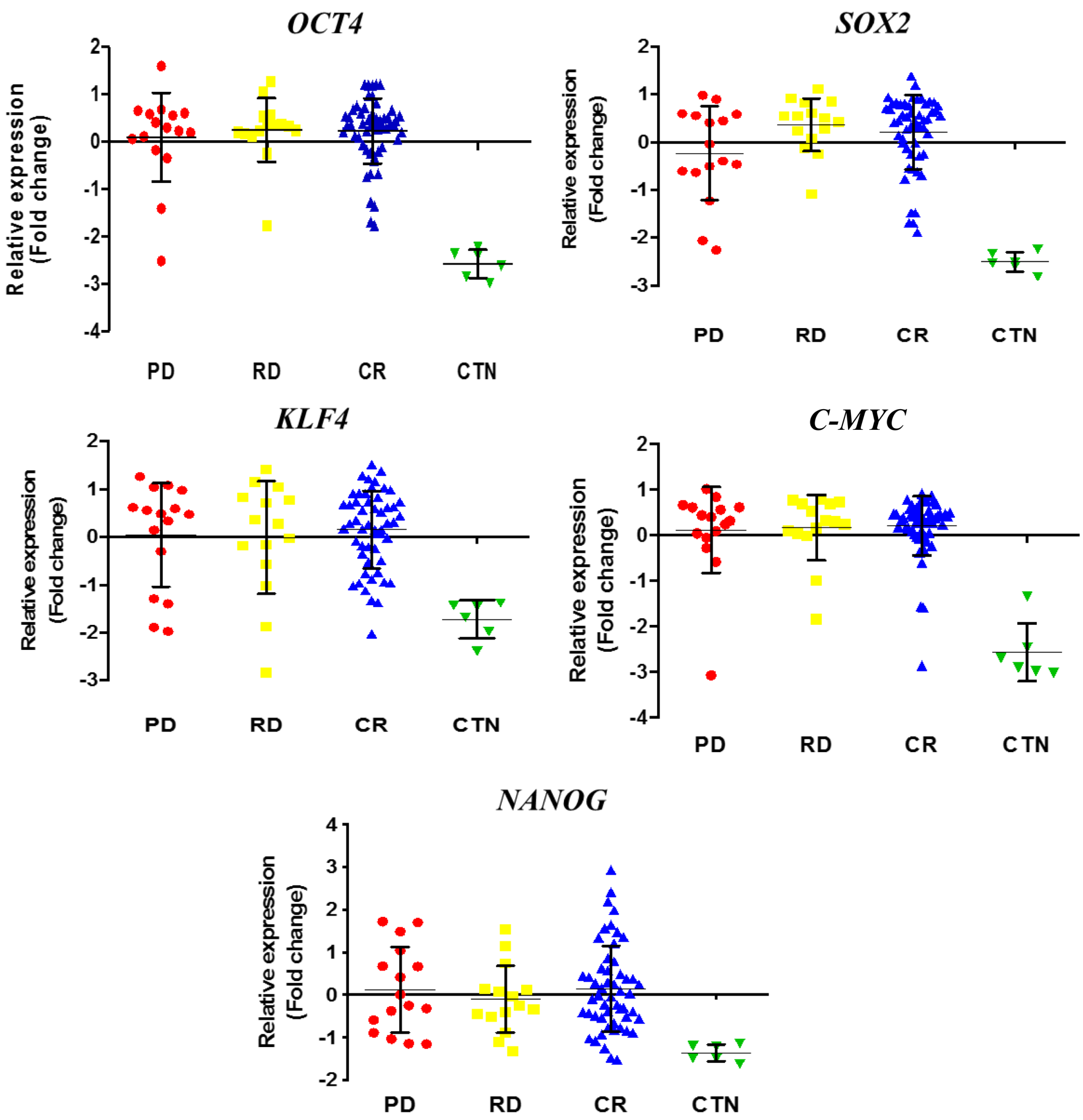

Figure S1. Expression of OSKM-N by clinical outcome. The scatter plots illustrate data from 85 cervical cancer patients grouped by clinical outcome. The gene intensity values were obtaining by microarray analyses for OCT4, SOX2, KLF4, C-MYC and NANOG. Patients were grouped by clinical outcome after five years of treatment. In red we show progressive disease (PD) group, yellow recurrent disease (RD), blue complete response (CR) and green cervical normal tissue (CNT).

Figure S2.

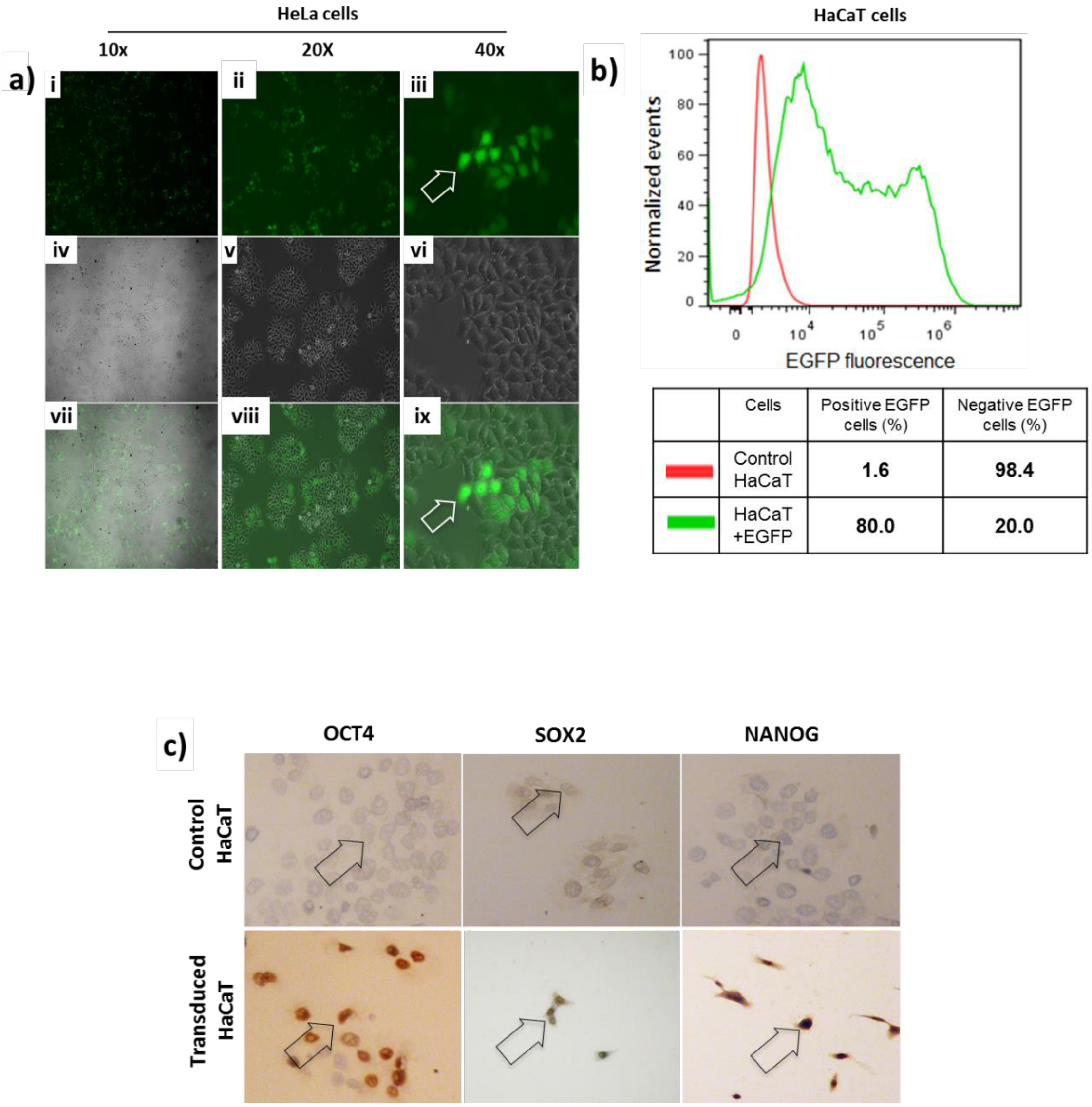

	Cells	Positive EGFP cells (%)	Negative EGFP cells (%)
(red)	Control HaCaT	**1.6**	**98.4**
(green)	HaCaT +EGFP	**80.0**	**20.0**

Figure S2. Infective lentiviruses transduce EGFP in HeLa and HaCaT cells. a) The lentiviral particles were infective in HeLa and HaCaT cells. a) EGFP fluorescence in HeLa cells was identified by microscopy and the arrows show the positive cells. b) 80 % of the population was positive EGFP fluorescence in HaCaT cells by flow cytometry. c) OCT4, SOX2 and NANOG factors were transduced in HaCaT cells and, after a period of selection with Puromycin, these factors were overexpressed and located in the nucleus as it is shown in the images by immunocytochemistry. Images acquired with a 40X magnification.

Figure S3

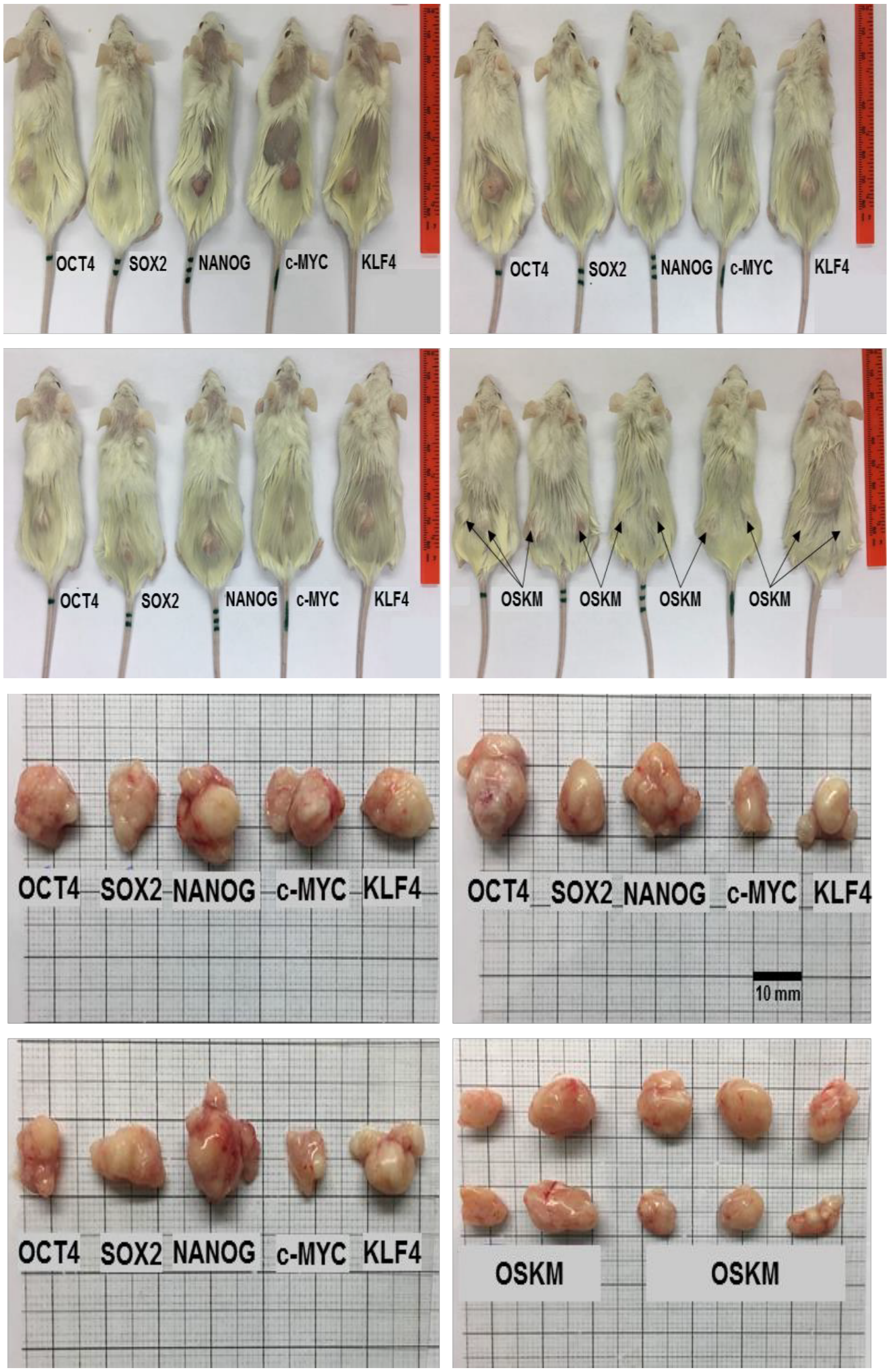

Figure S3. OSKM-N genes induce tumorigenic capacity in HaCaT cells. Representative images of tumor formed by $6x10^6$ cells of HaCaT transduced with OCT4, SOX2, NANOG, C-MYC, KLF4 and OSKM (OCT4, SOX2, KLF4 and C-MYC). HaCaT cells did not form a tumor with $8x10^6$ cells.

Figure S4

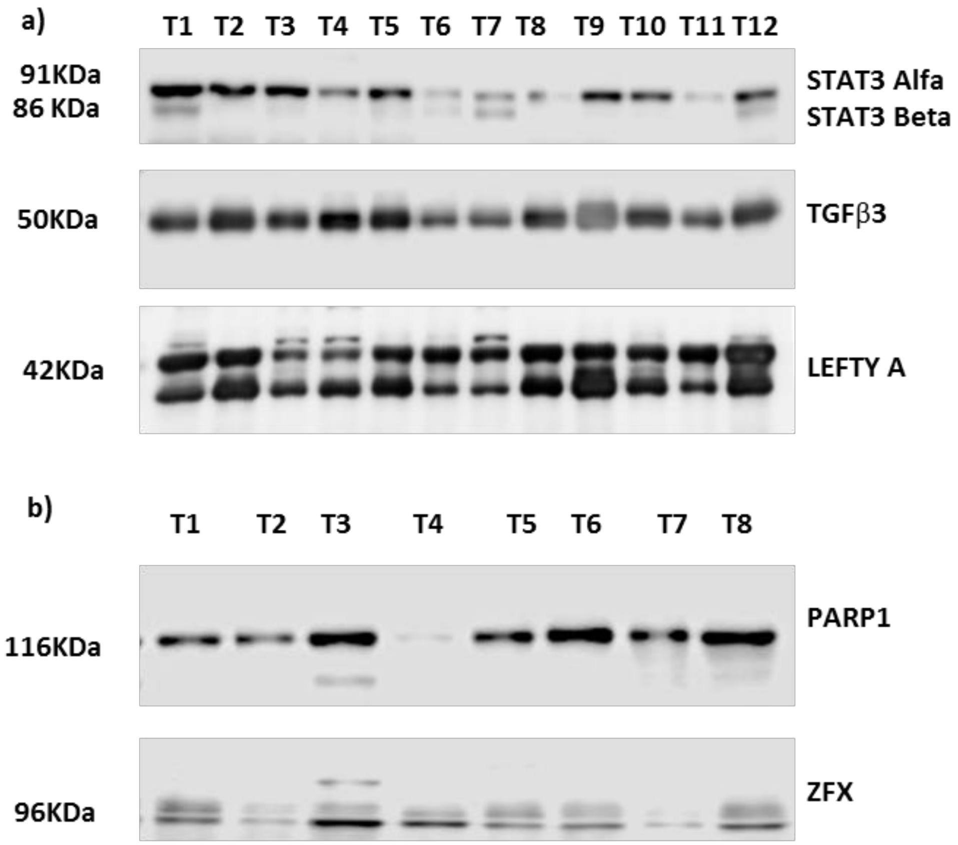

Figure S4. Genes associated with OSKM-N factors in cervical cancer. a) STAT3, TGFβ3 and LEFTY A proteins were detected in 12 cervical tumor samples and PARP1 and ZFX in 8 cervical tumor samples by western blot. Their presence in the cervical cancer suggests that they are also involved in the process of malignancy.
